# Long-term results from modified sphincteroplasty in patients with traumatic sphincter injury: a retrospective study

**DOI:** 10.1590/1516-3180.2020.0467.02112020

**Published:** 2020-12-23

**Authors:** Mustafa Berkesoglu, Tahsin Colak, Mehmet Ozgur Turkmenoglu, Ismet Han, Ilter Kirmizi, Gokhan Giray Akgul, Ihsan Gunduz

**Affiliations:** I MD. Assistant Professor, Department of General Surgery, School of Medicine, Mersin University Hospital, Mersin, Turkey.; II MD. Professor, Department of General Surgery, Division of Colorectal Surgery, School of Medicine, Mersin University Hospital, Mersin, Turkey.; III MD. Associate Professor, Department of General Surgery, Division of Colorectal Surgery, School of Medicine, Mersin University Hospital, Mersin, Turkey.; IV MD. Surgical Gastroenterologist, Department of Gastrointestinal Surgery, Trabzon Training and Research Hospital, Trabzon, Turkey; V MD. Surgical Gastroenterologist, Department of Gastrointestinal Surgery, Aydin State Hospital, Aydin, Turkey.; VI MD. Surgical Oncologist, Department of Surgical Oncology, Ankara Gulhane Training and Research Hospital, Ankara, Turkey.; VII MD. Surgical Gastroenterologist, Department of Gastrointestinal Surgery, Tekirdag State Hospital, Tekirdag, Turkey.

**Keywords:** Anal canal, Fecal incontinence, Perineum, Surgery [subheading], Anal sphincter, Anal incontinence, Sphincteroplasty.

## Abstract

**BACKGROUND:**

The results from sphincteroplasty may worsen over time. Reseparation of the rectum and vagina/scrotum in conjunction with sphincteroplasty achieves good results. Improving the surgical effect of sphincteroplasty through perineal body reconstruction is crucial.

**OBJECTIVE:**

To evaluate the long-term results from anterior sphincteroplasty and perineal body reconstruction (modified sphincteroplasty) among patients with traumatic sphincter injury.

**DESIGN AND SETTING:**

Retrospective study among patients who underwent modified sphincteroplasty in a university hospital between January 2006 and December 2018. Fifty patients were evaluated in detail.

**METHODS:**

The following variables were evaluated: gender, age, additional disease status, time interval between trauma and surgery, surgical technique, duration of hospitalization, follow-up period after surgery, manometric values, electromyography results, magnetic resonance imaging scans, Wexner scores, satisfaction levels with surgery and surgical outcomes.

**RESULTS:**

The patients’ mean age was 44.6 ± 15.1 years. The median follow-up period was 62 months (range, 12-118). The mean Wexner scores preoperatively, postoperatively in first month (M1S) and at the time of this report (AAS) were 15.5 ± 3.2, 1.9 ± 3.15 and 3.9 ± 5.3, respectively. Although improvements in the patients’ mean Wexner scores became impaired over time, the postoperative Wexner scores were still significantly better than the preoperative Wexner scores (P = 0.001).

**CONCLUSION:**

Good or excellent results were obtained surgically among patients with traumatic sphincter injury. Performing perineal body reconstruction in addition to sphincteroplasty can provide better long-term continence. Surgical outcomes were found to be better, especially among patients younger than 50 years of age and among patients who underwent surgery within the first five years after trauma.

## INTRODUCTION

Continence is one of the main factors that determine the quality of life.^1^ Pelvic muscle groups, sphincter function, nervous system, rectal compliance, fecal contents and cognitive functions act towards maintaining continence.^2^ Fecal or anal incontinence (AI) may be defined as involuntary leakage of rectal contents or inability to delay defecation until an appropriate time.^1^ Incontinence causes social and psychological problems as well as medical and economic effects, adversely affects the quality of life and is generally kept secret by the patients.^3^^,^^4^^,^^5^^,^^6^

Although sphincter structure is preserved in non-traumatic situations such as diabetes mellitus and neurological disease, structural loss or weakness in the sphincters usually occurs through traumatic conditions such as anorectal surgeries, obstetric injuries and traumas^2^^,^^4^^,^^7^^,^^8^^,^^9^^,^^10^^,^^11^

Many surgical and non-surgical methods with different success rates may be preferred in treatments for AI. According to the severity of the complaints and the development mechanisms of the disease, AI can be managed conservatively through diet, antidiarrheal medicine and biofeedback.^4^ Surgical treatment is usually performed, when the anal sphincter has an anatomical defect or when conservative treatment is not successful.^12^^,^^13^ The success rates of various surgical techniques range from 25% to 90%.^14^^,^^15^ There is no consensus on which method is most effective for treating traumatic sphincter injury.^8^

Sphincteroplasty is the commonly preferred surgical treatment among the surgical options.^14^^,^^15^ It has the considerable advantage that it does not require any purchase of additional equipment or any cost.^1^^,^^4^^,^^16^^,^^17^ However, the results obtained from sphincteroplasty may become impaired over time. The patient’s age, cause of the injury, timing of surgery, timing of postoperative assessment and variation in surgical techniques are among the factors that may affect the success of sphincteroplasty.^4^^,^^5^^,^^9^^,^^12^^,^^18^ Reseparation of the rectum and the vagina/scrotum in conjunction with sphincteroplasty achieves good results, and performing perineal body reconstruction in addition to sphincteroplasty can provide better long-term continence.^19^

## OBJECTIVE

In this study, we aimed to evaluate the long-term results from anterior sphincteroplasty and perineal body reconstruction (modified sphincteroplasty) among patients with traumatic sphincter injuries.

## METHODS

This study was approved by our university’s local ethics committee (number: 2018/203). Seventy-four surgical patients with AI who were seen between January 2006 and December 2018 were evaluated retrospectively. Patients older than 65 years of age with poor health status (n = 4), patients with past surgical history due to AI (n = 2), patients with multiple sphincter injuries (n = 3), patients with inflammatory bowel disease (n = 3), patients with neurological disease (n = 7), patients with diabetes mellitus (n = 2) and patients with lack of data (n = 7) were excluded from the study. A total of 50 patients who underwent modified sphincteroplasty were evaluated in detail.

The sphincter defect was evaluated preoperatively by means of pelvic magnetic resonance imaging (MRI), in all patients. Early reconstruction was defined as ‘surgery performed within 14 days after the trauma’. A preoperative evaluation using anal manometry and anal electromyography (EMG) was performed in the cases of patients without early reconstruction. Patients with AI following vaginal delivery had grade 3 injuries (involving partial or complete disruption of the anal sphincter complex, i.e. including the external anal sphincter and the internal anal sphincter) and grade 4 injuries (involving disruption of the anal mucosa in addition to the sphincter complex).^20^

The patients’ AI scores were evaluated using the Cleveland Clinic Florida Incontinence Scoring Scale (Wexner score) preoperatively, postoperatively in the first month (M1S) and at the time of this report (AAS). The assessment comprised five items: frequency of AI (for solid, liquid and gas components), wearing of pads and lifestyle alteration due to AI.^21^ The total score (range: 0-20) was calculated by summing the ‘0-4’ points for each parameter (0 points for complete continence and 20 points for complete incontinence, according to the Wexner score).

In addition, the patients’ satisfaction was evaluated using the Cleveland Clinical Quality of Life Score, on a scale of 1-10 points (1 point, ‘lowest’ satisfaction score of patient; 10 points, ‘highest’ satisfaction score relating to surgery).

The outcome was classified as excellent (full continence), good (incontinence in relation to flatus or sporadic loss of liquid stool was encountered postoperatively, after less than one month); moderate (incontinence was regularly experienced in relation to liquid/solid stools, but incontinence episodes were reduced by 50% or more postoperatively); or poor (persistent AI with less than 50% reduction of incontinence episodes postoperatively).^15^ All the data were obtained using medical record and interviews.

### Intervention techniques

**Figure 1.**
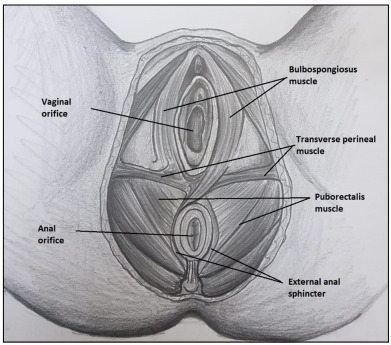
The final aim of reconstruction to obtain continence.

Bowel preparation and urinary drainage with a catheter were not routinely performed. Antibiotic prophylaxis was administered, consisting of 1 g of cefazolin and 500 mg of metronidazole. The operations were performed in the lithotomy position by the same surgical team (TC, MOT). The final aim of the surgery was to perform reconstruction of the pelvic floor for continence (Figure 1).

**Figure 2.**
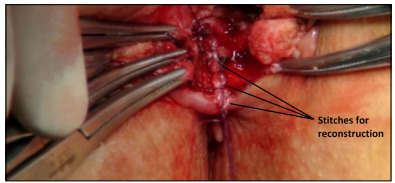
Reconstruction of sphincter muscles in patient with traumatic sphincter injury.

Anterior sphincteroplasty was performed under direct vision. A curvilinear incision was made in the perianal area and the dissection was completed with preservation of the rectal wall. Both sides of the puborectalis muscles were dissected until the mesorectum tissue appeared. We attempted to preserve the pudendal nerves by avoiding excessive dissection laterally. Subsequently, bulbospongiosus muscles were introduced and the tissue layers (puborectalis muscles, external sphincter muscles, internal anal sphincter muscles and mucosa) were sutured at the midline, from deep to superficial, using 2-0 delayed absorbable polyglactin suture material (Vicryl, Ethicon Inc, NJ, United States) (Figure 2).

**Figure 3.**
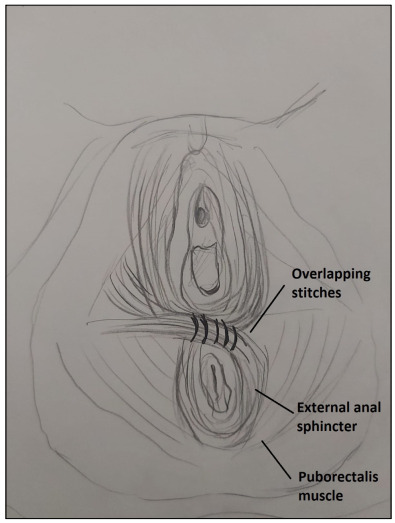
Illustration of overlapping sphincteroplasty.

**Figure 4.**
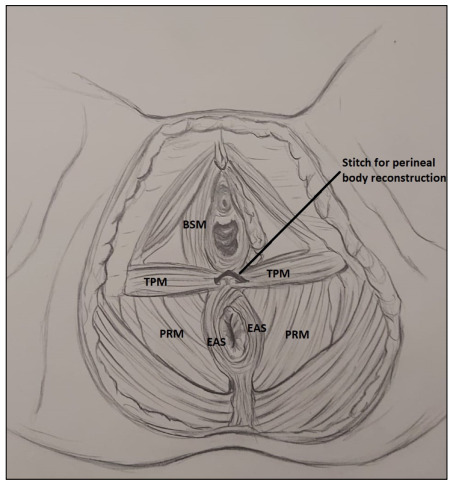
Reconstruction of perineal body with stitch.

**Figure 5.**
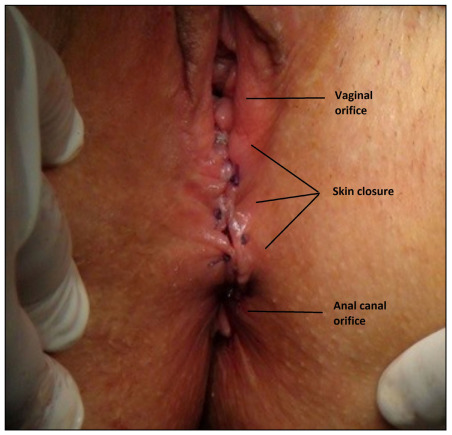
Final view: reseparation of rectum and vagina.

The external anal sphincters were mainly reconstructed using an overlapping technique (Figure 3). In most cases, overlapping sphincter reconstruction was performed by attempting to preserve muscle mass without removing the fibrotic area at the median line. Lastly, the bulbospongiosus muscles were reconstructed, and the posterior part of the bulbospongiosus muscles, median edge of the transverse perineal muscles, anterior part of the puborectalis muscles and anterior part of the external anal sphincter muscles were combined in the anterior part to reconstruct the perineal body (Figure 4). We aimed to create a circular muscle mass around the anal canal. Surgical drains were not used routinely (Figure 5). According to the status of the perineal injury, a temporary stoma was provided for fecal diversion.

BSM = bulbospongiosus muscle; TPM = transverse perineal muscle; PRM = puborectalis muscle; EAS = external anal sphincter muscle.

The patients were discharged from the hospital after bowel movements had become reestablished. They were evaluated postoperatively after the first week, first month, third month, sixth month, first year and at the time of making the current report.

### Statistical analysis

The patients were evaluated regarding gender, age, additional disease status, time interval between trauma and surgery, surgical technique, duration of hospitalization, follow-up period after surgery, manometric values, EMG results, MRI scans, Wexner scores, satisfaction levels with surgery and surgical outcomes. Variables were presented as percentages (%), means ± standard deviations (SD) and medians (minimum-maximum). Categorical variables were evaluated using the chi-square test. Continuous variables were assessed using one-way ANOVA. Groups were compared using the chi-square test or Mann-Whitney U test. Wexner scores were compared between different time points by means of ANOVA. The statistical analysis was performed using the SPSS 15.0 software (SPSS, Chicago, IL, United States) and P < 0.05 was considered statistically significant.

## RESULTS

Out of the total of 50 patients evaluated in our study (Table 1), most of them were women (72%). The major cause of traumatic sphincter injury was vaginal delivery (54%) in this study.

**Table 1.** Results from the patients and surgery

	n (%)	Mean ± standard deviation (SD)
Sex		
Women	36 (72)	
Men	14 (28)	
Etiology		
Vaginal delivery	27 (54)	
Anorectal surgery	12 (24)	
Nonsurgical trauma (any other trauma or abuse)	11 (22)	
Surgery		
Overlapping sphincteroplasty	41 (82)	
End-to-end sphincteroplasty	9 (18)	
Surgery		
Early reconstruction (within 14 days)	16 (32)	
Elective surgery	34 (68)	
Mean age		44.6 ± 15.1 years
Interval between injury and surgery		5.6 ± 8.2 years
Mean Wexner score		
Preoperative		15.5 ± 3.2
First postoperative month		1.92 ± 3.15
At the time of this report		3.9 ± 5.3

All the patients underwent modified sphincteroplasty; overlapping sphincteroplasty was performed in the majority of the cases (82%). Eight patients with perineal injuries following traffic accidents and other blunt trauma underwent simultaneous modified sphincteroplasty and fecal diversion with loop colostomy. The mean duration of closure of the stoma was 9.5 ± 2.8 months. The mean duration of hospitalization was 4.0 ± 4.6 days, and no mortality was detected. Eleven patients developed postoperative wound infection and four patients had wound dehiscence. No patient underwent additional surgery for postoperative wound complications. The median follow-up period was 62 months (range: 12-118).

Manometric measurements were obtained from 54% of the patients (n = 27), both preoperatively and postoperatively. The preoperative measured mean resting pressure became elevated in the postoperative period (respectively, 52.11 ± 17.55 cmH_2_O and 57.37 ± 16.26 cmH_2_O; P = 0.2585). The preoperative measured mean squeeze pressure also became elevated in the postoperative period (respectively, 93.63 ± 33.56 cmH_2_O and 113 ± 35.03 cmH_2_O; P = 0.037).

The mean preoperative Wexner score was higher than the mean M1S Wexner score and mean AAS Wexner score. Although the improvement in mean Wexner score decreased over time, it was still better than the preoperative values (preoperative versus M1S, P < 0.001; preoperative versus AAS, P < 0.001; M1S versus AAS, P = 0.024).

Among the 50 patients evaluated here, 32 (64%) were younger than 50 years of age. There was a statistically significant difference in mean AAS Wexner score between the age groups (younger or older than 50 years), and improvements in mean AAS Wexner score were obtained among the patients younger than 50 years of age (P = 0.024).

Out of 36 patients who were operated within the first five years after their sphincter injury, 16 patients underwent early reconstruction. Although there was no statistically significant difference in AAS Wexner score for patients who underwent early reconstruction, statistically significant better results were obtained among the patients who underwent surgery within first five years after the trauma (P = 0.031).

Continence of gases, fluids and solids was achieved at M1S, respectively in 72% (n = 36), 86% (n = 43) and 92% (n = 46) of the patients after the modified sphincteroplasty. However, the continence rates for gases, fluids among these patients at AAS were, respectively, 64% (n = 32), 74% (n = 37) and 84% (n = 42). The postoperative improvement in continence status remained stable in 36 patients during the follow-up period, while the continence status of the other 14 patients deteriorated to varying degrees over time. Good or excellent continence was obtained in 84% of patients at AAS.

There was no statistically significant difference in AAS Wexner score or in patients’ satisfaction with surgery, between the overlapping and end-to-end modified sphincteroplasty groups (P > 0.05 for both). The mean score regarding satisfaction with surgery was 8.1 ± 2.5. Although satisfaction with surgery was perfect (10 points) in 42% of the patients (n = 21), the level of satisfaction with surgery was below 5 points in 10% of the patients (n = 5). Lower AAS Wexner scores correlated with greater satisfaction with surgery.

## DISCUSSION

All the patients in this study presented traumatic anal sphincter injury. Modified sphincteroplasty was performed in all cases, and the levels of satisfaction with surgery remained high over the long term. Incontinence scores were lower among patients younger than 50 years of age and among patients who underwent surgery within the first five years after the trauma.

Incontinence is a symptom of varying severity that can range from mild leakage of gas to complete loss of fecal control. It has been reported that its prevalence generally ranges from 1% to 21%, although it is seen more frequently among individuals over the age of 65 years.^2^ Different incidence rates may be associated with variations in the definition of “incontinence”. ‘Anorectal incontinence’, ‘AI’ and ‘fecal incontinence’ may be used to describe incontinence.^2^^,^^4^^,^^7^^,^^8^^,^^9^ While uncontrolled leakage of gases and stools is defined as AI,^4^ fecal incontinence usually describes leakage of stools.^8^ Diarrhea, neurological diseases, surgical or obstetrical trauma and advanced age are among the causes of AI.^7^ Primary incontinence may be seen as a result of congenital diseases, and secondary incontinence may be seen in acquired cases.^2^

The most common etiological reason for referrals to colorectal surgeons is traumatic sphincter injury, among the many different cases. It is crucial to obtain detailed anamnesis of each case. However, it needs to be borne in mind that some patients may tend to respond incorrectly during questioning to obtain the detailed past medical history.

During the physical examination, the perineal region and anoderm is evaluated for any tears or leakage. Detailed evaluation is crucial for differentiating leakage from AI in diseases such as rectal prolapsus.^12^^,^^15^^,^^16^^,^^17^ It is important to recognize the type, frequency and extent of AI. Scoring systems have been developed for evaluation of AI. One of the most widely used scales is the Wexner scoring system.^21^ All of the patients in the present study presented acquired traumatic sphincter injuries, and the Wexner scoring systems were used for assessment of AI at different times among these patients.

Diagnostic endoanal ultrasonography (USG), anorectal manometry, pudendal nerve evaluation, EMG, defecography and colonoscopy may be performed.^2^ Predictions from preoperative manometric measurements may yield conflicting outcomes.^9^^,^^18^^,^^22^ In manometric measurements made in the present study, there was no significant postoperative result regarding the mean resting pressure, which was more related to internal anal sphincters. Statistically significant results were obtained with regard to mean squeeze pressures postoperatively, which were associated with external sphincter functions and voluntary contraction.

MRI identifies the structure of the external anal sphincters and puborectalis muscles, and also the pelvic floor structure.^13^ We mainly used MRI for our evaluations; USG is valuable but technically difficult, especially among patients with severe trauma in the early period.

Biofeedback, dietary recommendations, regulation of medications, arrangement of fibers and use of medical drugs can reduce the symptoms of AI.^2^^,^^17^ Sphincteroplasty is one of the most preferred treatments, especially among patients with anatomical sphincter defects after trauma.^3^^,^^4^^,^^5^^,^^17^^,^^23^ Especially among patients with traumatic sphincter injury, anterior sphincteroplasty may reduce their complaints, and the overlapping technique should be the first surgical option among different forms of sphincteroplasty.^2^^,^^12^^,^^15^^,^^16^^,^^17^

Artificial sphincter applications, sacral nerve stimulation, graciloplasty, anal encircling methods, tibial nerve stimulation, Secca^®^ procedure (Curon Medical, Inc., Fremont, CA, United States), gluteoplasty and antegrade enema applications are other surgical treatment options in addition to sphincteroplasty.^4^^,^^5^^,^^17^ However, it should be noted that most of these methods are expensive due to their use of additional implants during the procedure.^4^ The short-term success rates of different types of treatment have been reported to range from 31% to 83%.^4^^,^^16^^,^^17^

Over long-term follow-up, decreasing success rates have previously been reported.^1^^,^^17^ Sphincteroplasty has been reported to yield short-term improvement (68-74%) in AI, but the success rate may decline to 0-50% over time.^1^^,^^4^^,^^12^^,^^16^^,^^24^ Damage to the distal branches of the pudendal nerve during surgery, variation in surgical technique, suture breakage and muscle denervation with age are some of the possible causes of this deterioration over the long term.^16^^,^^25^

Despite this deterioration of continence, patients may feel satisfied with their surgical outcomes and quality of life.^17^ Lehto et al. reported improvement in both fecal incontinence and quality of life among patients in all age groups after sphincteroplasty.^22^ However younger patients (< 50 years old) had better surgical outcomes than older ones.^9^^,^^15^^,^^22^ In the present study, the postoperative mean AAS Wexner score of younger patients (18-50 years old) was also significantly better than those of patients older than 50 years (P = 0.024).

Some studies have pointed out that biofeedback treatment or reoperation might be useful for preserving the good results.^16^^,^^25^^,^^26^ Modified sphincteroplasty was performed on all patients in this study. Significant improvement in continence was observed in most of the patients during the long follow-up period (median of 62 months; range, 12-118 months). Similar or worse outcomes after sphincteroplasty were reported in some other studies with long follow-up periods (60 months or longer).^4^^,^^12^^,^^17^^,^^24^ It was considered that the possible reasons for a successful outcome in the present study may have related to the surgical technique and the patient group selected, such as those with traumatic sphincter injury.

In the present study, overlapping sphincteroplasty was performed in the majority of cases (82%). Perineal body reconstruction was performed in all cases and lateral dissection was limited during the surgery. According to our experience, strengthening of all the available functional muscles, restoration of normal anatomy and reseparation of the rectum and vagina/scrotum in conjunction with use of modified sphincteroplasty increased the success rate. It has been emphasized that, especially in cases of high-grade obstetric injuries, reconstruction should be performed by an expert and specialist team during the early period.^20^ In the present study, modified sphincteroplasty was performed always by same expert surgical team. Although better results were not obtained among patients who underwent early reconstruction (P = 0.308), patients who underwent modified sphincteroplasty within five years after the trauma had better results than did those whose surgery was performed later.

There are some limitations to our study. Firstly, this was a retrospective study, and endoanal USG and manometry data were not obtained in all cases. Secondly, the results were not compared among the etiological subgroups in detail because of the small sample size. On the other hand, the study population was homogenous and long-term follow-up was obtained.

## CONCLUSION

Good or excellent results were obtained through use of modified sphincteroplasty, especially in treating traumatic sphincter injury. The surgical outcomes were found to be better among patients younger than 50 years of age and among patients who underwent surgery within the first five years after their trauma. It is crucial to recognize patients with AI and to direct them to centers with reconstruction experience, without delay. Lastly, perineal body reconstruction may provide long-term preservation of improvement when combined with anterior sphincteroplasty, among patients with traumatic AI.

## References

[1] Ruiz NS, Kaiser AM (2017). Fecal incontinence - Challenges and solutions. World J Gastroenterol.

[2] Alavi K, Chan S, Wise P (2015). Fecal Incontinence: Etiology, Diagnosis, and Management. J Gastrointest Surg.

[3] Hayden DM, Weiss EG (2011). Fecal incontinence: etiology, evaluation, and treatment. Clin Colon Rectal Surg.

[4] Pescatori LC, Pescatori M (2014). Sphincteroplasty for anal incontinence. Gastroenterol Rep (Oxf).

[5] Mitchell PJ, Sagar PM (2014). Emerging surgical therapies for faecal incontinence. Nat Rev Gastroenterol Hepatol.

[6] Mellgren A (2010). Fecal incontinence. Surg Clin North Am.

[7] Andromanakos NP, Filippou DK, Pinis SI, Kostakis AI (2013). Anorectal incontinence: a challenge in diagnostic and therapeutic approach. Eur J Gastroenterol Hepatol.

[8] Brown SR, Wadhawan H, Nelson RL (2013). Surgery for faecal incontinence in adults. Cochrane Database Syst Rev.

[9] Matzel KE, Bittorf B (2016). Management of fecal incontinence. Seminars in Colon and Rectal Surgery.

[10] Oberwalder M, Connor J, Wexner SD (2003). Meta-analysis to determine the incidence of obstetric anal sphincter damage. Br J Surg.

[11] Ng KS, Sivakumaran Y, Nassar N, Gladman MA (2015). Fecal Incontinence: Community Prevalence and Associated Factors--A Systematic Review. Dis Colon Rectum.

[12] Mevik K, Norderval S, Kileng H, Johansen M, Vonen B (2009). Long-term results after anterior sphincteroplasty for anal incontinence. Scand J Surg.

[13] Bharucha AE, Fletcher JG, Melton LJ, Zinsmeister AR (2012). Obstetric trauma, pelvic floor injury and fecal incontinence: a population-based case-control study. Am J Gastroenterol.

[14] Meurette G, Duchalais E, Lehur PA (2014). Surgical approaches to fecal incontinence in the adult. J Visc Surg.

[15] Oom DM, Gosselink MP, Schouten WR (2009). Anterior sphincteroplasty for fecal incontinence: a single center experience in the era of sacral neuromodulation. Dis Colon Rectum.

[16] Altomare DF, De Fazio M, Giuliani RT, Catalano G, Cuccia F (2010). Sphincteroplasty for fecal incontinence in the era of sacral nerve modulation. World J Gastroenterol.

[17] Bleier JI, Kann BR (2013). Surgical management of fecal incontinence. Gastroenterol Clin North Am.

[18] Chase S, Mittal R, Jesudason MR, Nayak S, Perakath B (2010). Anal sphincter repair for fecal incontinence: experience from a tertiary care centre. Indian J Gastroenterol.

[19] Ogilvie JW, Madoff RD, Altomore DF, Ratto C, Doglietto GB (2007). Sphincteroplasty. Fecal incontinence diagnosis and treatment.

[20] Harvey MA, Pierce M, Alter JE (2015). Obstetrical Anal Sphincter Injuries (OASIS): Prevention, Recognition, and Repair. J Obstet Gynaecol Can.

[21] Vaizey CJ, Carapeti E, Cahill JA, Kamm MA (1999). Prospective comparison of faecal incontinence grading systems. Gut.

[22] Lehto K, Hyöty M, Collin P, Huhtala H, Aitola P (2013). Seven-year follow-up after anterior sphincter reconstruction for faecal incontinence. Int J Colorectal Dis.

[23] McManus BP, Allison S, Hernánchez-Sánchez J (2015). Anterior sphincteroplasty for fecal incontinence: predicting incontinence relapse. Int J Colorectal Dis.

[24] Barisic GI, Krivokapic ZV, Markovic VA (2006). Outcome of overlapping anal sphincter repair after 3 months and after a mean of 80 months. Int J Colorectal Dis.

[25] Malouf AJ, Norton CS, Engel AF, Nicholls RJ, Kamm MA (2000). Long-term results of overlapping anterior anal-sphincter repair for obstetric trauma. Lancet.

[26] Pinus J, Martins JL (1997). Use of biofeedback (BFB) in the treatment of fecal incontinence after surgical correction of anorectal malformations by sagittal anorectoplasty (PSARP). Sao Paulo Med J.

